# 1,7,8,9,10,10-Hexachloro-4-(2-phenyl­eth­yl)-4-aza­tricyclo­[5.2.1.0^2,6^]dec-8-ene-3,5-dione

**DOI:** 10.1107/S1600536811022495

**Published:** 2011-06-18

**Authors:** R. Manohar, M. Harikrishna, C. R. Ramanathan, M. SureshKumar, K. Gunasekaran

**Affiliations:** a CAS in Crystallography and Biophysics, University of Madras, Guindy Campus, Chennai 600 025, India; b Department of Chemistry, Pondicherry University, Pondicherry 605 014, India; c Centre for Bioinformatics, Pondicherry University, Pondicherry 605 014, India

## Abstract

In the title compound, C_17_H_11_Cl_6_NO_2_, the six-membered ring of the norbornene moiety adopts a boat conformation whereas the two five-membered rings adopt envelope conformations. The phenyl ring and the ring of the succinimide moiety are almost coplanar [dihedral angle = 7.44 (14)°]. The crystal packing is stabilized by a weak inter­molecular C—H⋯O hydrogen bond.

## Related literature

The inter­est in cyclic imides is due to their biological activity and wide application in the pharmaceutical industry, see: Duarte *et al.* (2006 ▶); Nakamura *et al.* (2006 ▶); Stefańska *et al.* (2010 ▶).
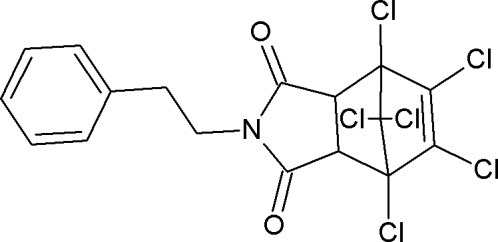


## Experimental

### 

#### Crystal data

C_17_H_11_Cl_6_NO_2_
*M* *_r_* = 473.97
Monoclinic, 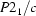

*a* = 13.3009 (5) Å
*b* = 13.6141 (5) Å
*c* = 11.4912 (4) Å
β = 111.276 (4)°
*V* = 1939.00 (12) Å^3^
*Z* = 4
Mo *K*α radiation
μ = 0.90 mm^−1^
*T* = 293 K
0.20 × 0.20 × 0.20 mm

#### Data collection

Oxford Diffraction Xcalibur Eos diffractometer
Absorption correction: multi-scan (*CrysAlis PRO*; Oxford Diffraction, 2010 ▶) *T*_min_ = 0.978, *T*_max_ = 0.984
9237 measured reflections
4457 independent reflections
2814 reflections with *I* > 2σ(*I*)
*R*_int_ = 0.021

#### Refinement

*R*[*F* ^2^ > 2σ(*F* ^2^)] = 0.042
*wR*(*F* ^2^) = 0.121
*S* = 0.72
4457 reflections
244 parameters
H atoms treated by a mixture of independent and constrained refinement
Δρ_max_ = 0.49 e Å^−3^
Δρ_min_ = −0.54 e Å^−3^

### 

Data collection: *CrysAlis PRO* (Oxford Diffraction, 2010 ▶); cell refinement: *CrysAlis PRO*; data reduction: *CrysAlis PRO*; program(s) used to solve structure: *SHELXS97* (Sheldrick, 2008 ▶); program(s) used to refine structure: *SHELXL97* (Sheldrick, 2008 ▶); molecular graphics: *ORTEP-3* (Farrugia, 1997 ▶); software used to prepare material for publication: *SHELXL97* and *PLATON* (Spek, 2009 ▶).

## Supplementary Material

Crystal structure: contains datablock(s) global, I. DOI: 10.1107/S1600536811022495/bt5543sup1.cif

Structure factors: contains datablock(s) I. DOI: 10.1107/S1600536811022495/bt5543Isup2.hkl

Supplementary material file. DOI: 10.1107/S1600536811022495/bt5543Isup3.cml

Additional supplementary materials:  crystallographic information; 3D view; checkCIF report

## Figures and Tables

**Table 1:** Hydrogen-bond geometry (Å, °)

*D*—H⋯*A*	*D*—H	H⋯*A*	*D*⋯*A*	*D*—H⋯*A*
C2—H1⋯O2^i^	0.91 (3)	2.50 (3)	3.235 (3)	138 (2)

Symmetry code: (i) 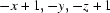.

**Fig. 1.**
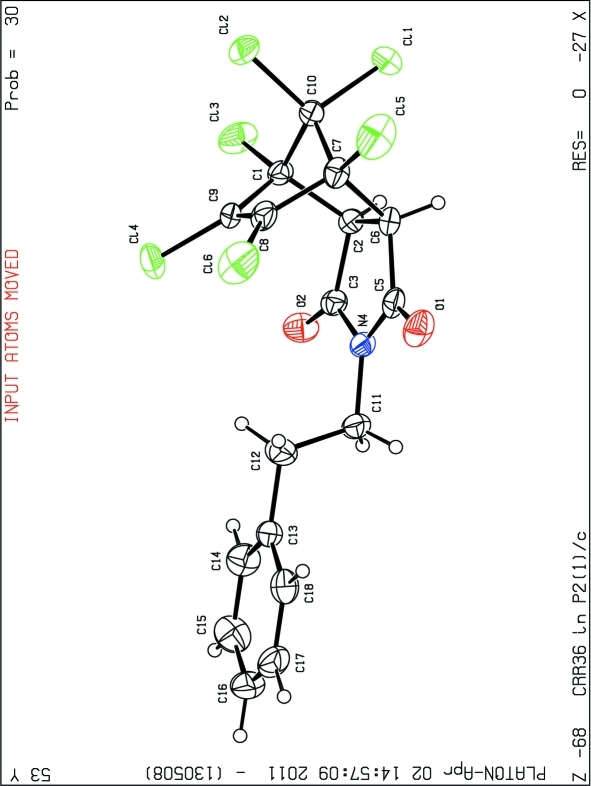
The ORTEP diagram of the compound with 30% probability displacement ellipsoids.

**Fig. 2.**
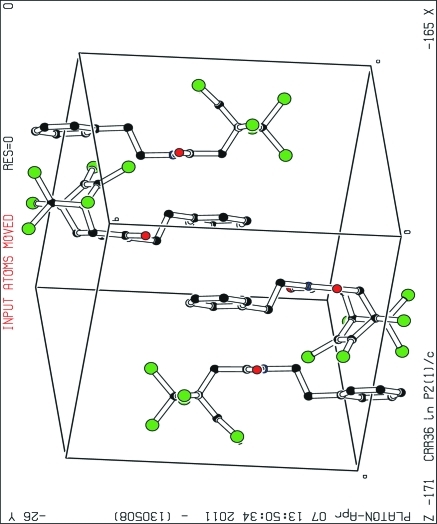
Packing diagram.

## supplementary crystallographic information

### Comment

The *ORTEP* diagram for the molecule of the title compound is given in Fig. 1. In the crystal structure, the phenyl and the azatricyclo substitutions are in anti conformation about the C11—C12 bond which is confirmed by the torsion angle N4—C11—C12—C13 [169.3 (3)°]. In the crystal, molecules are linked by weak intermolecular C—H···O hydrogen bonds.

### Experimental

Phenethylamine (1 equiv) and 1,4,5,6,7,7-hexachloro-5-norbornene-2,3-dicarboxylic anhydride (1 equiv) were stirred at room temperature in dry ethyl acetate for 30 min. Ethyl acetate was removed under reduced pressure; the resulting residue was dissolved in toluene. To this reaction mixture was added acetyl chloride (5 equiv) and refluxed for 1 h. The reaction mixture was brought to room temperature and washed with aqueous Na2CO3 and dried over anhydrous Na_2_SO_4_. Filtered and concentrated under reduced pressure followed by silica gel column purification afforded the imide,1,7,8,9,10,10-Hexachloro-4-(2-phenylethyl)-4-azatricyclo [5.2.1.02,6]dec-8-ene-3,5-dione,in 55% yield as colourless solid (m.p.: 161–163°C).

### Refinement

The hydrogen atoms at ring fusion were located using difference Fourier map and refined isotropically. Other H atoms were positioned geometrically with C-H = 0.93Å for aromatic H and C-H = 0.97å for methylene H and refined using a riding model with U(H) set 1.2 U_eq_(C).

### Crystal data

**Table:**

C_17_H_11_Cl_6_NO_2_	*Z* = 4
*M**_r_* = 473.97	*F*(000) = 952
Monoclinic, *P*2_1_/*c*	*D*_x_ = 1.624 Mg m^−^^3^
Hall symbol: -P 2ybc	Mo *K*α radiation, λ = 0.71073 Å
*a* = 13.3009 (5) Å	θ = 3.0–29.3°
*b* = 13.6141 (5) Å	µ = 0.90 mm^−^^1^
*c* = 11.4912 (4) Å	*T* = 293 K
β = 111.276 (4)°	Monoclinic, colourless
*V* = 1939.00 (12) Å^3^	0.20 × 0.20 × 0.20 mm

### Data collection

**Table:**

Oxford Diffraction Xcalibur Eos diffractometer	4457 independent reflections
Radiation source: fine-focus sealed tube	2814 reflections with *I* > 2σ(*I*)
graphite	*R*_int_ = 0.021
Detector resolution: 15.9821 pixels mm^-1^	θ_max_ = 29.3°, θ_min_ = 3.0°
ω scans	*h* = −17→17
Absorption correction: multi-scan (*CrysAlis PRO*; Oxford Diffraction, 2010)	*k* = −17→14
*T*_min_ = 0.978, *T*_max_ = 0.984	*l* = −15→8
9237 measured reflections	

### Refinement

**Table:**

Refinement on *F*^2^	Secondary atom site location: difference Fourier map
Least-squares matrix: full	Hydrogen site location: inferred from neighbouring sites
*R*[*F*^2^ > 2σ(*F*^2^)] = 0.042	H atoms treated by a mixture of independent and constrained refinement
*wR*(*F*^2^) = 0.121	*w* = 1/[σ^2^(*F*_o_^2^) + (0.0784*P*)^2^ + 2.150*P*] where *P* = (*F*_o_^2^ + 2*F*_c_^2^)/3
*S* = 0.72	(Δ/σ)_max_ < 0.001
4457 reflections	Δρ_max_ = 0.49 e Å^−^^3^
244 parameters	Δρ_min_ = −0.54 e Å^−^^3^
0 restraints	Extinction correction: *SHELXL97* (Sheldrick, 2008), Fc^*^=kFc[1+0.001xFc^2^λ^3^/sin(2θ)]^-1/4^
Primary atom site location: structure-invariant direct methods	Extinction coefficient: 0.0027 (7)

### Special details

**Table:**

Geometry. All e.s.d.'s (except the e.s.d. in the dihedral angle between two l.s. planes) are estimated using the full covariance matrix. The cell e.s.d.'s are taken into account individually in the estimation of e.s.d.'s in distances, angles and torsion angles; correlations between e.s.d.'s in cell parameters are only used when they are defined by crystal symmetry. An approximate (isotropic) treatment of cell e.s.d.'s is used for estimating e.s.d.'s involving l.s. planes.
Refinement. Refinement of *F*^2^ against ALL reflections. The weighted *R*-factor *wR* and goodness of fit *S* are based on *F*^2^, conventional *R*-factors *R* are based on *F*, with *F* set to zero for negative *F*^2^. The threshold expression of *F*^2^ > σ(*F*^2^) is used only for calculating *R*-factors(gt) *etc*. and is not relevant to the choice of reflections for refinement. *R*-factors based on *F*^2^ are statistically about twice as large as those based on *F*, and *R*- factors based on ALL data will be even larger.

### Fractional atomic coordinates and isotropic or equivalent isotropic displacement parameters (Å^2^)

**Table:**

	*x*	*y*	*z*	*U*_iso_*/*U*_eq_	
C1	0.2561 (2)	0.08000 (19)	0.4510 (2)	0.0363 (5)	
C2	0.31543 (19)	0.02382 (19)	0.3771 (2)	0.0333 (5)	
C3	0.36261 (19)	−0.0735 (2)	0.4348 (2)	0.0379 (6)	
C5	0.2247 (2)	−0.1141 (2)	0.2485 (2)	0.0415 (6)	
C6	0.22344 (19)	−0.0031 (2)	0.2548 (2)	0.0359 (6)	
C7	0.1224 (2)	0.0410 (2)	0.2729 (2)	0.0418 (6)	
C8	0.1055 (2)	−0.0167 (2)	0.3772 (3)	0.0460 (7)	
C9	0.1842 (2)	0.0067 (2)	0.4824 (2)	0.0428 (6)	
C10	0.1697 (2)	0.1367 (2)	0.3446 (2)	0.0399 (6)	
C11	0.3368 (3)	−0.2506 (2)	0.3813 (3)	0.0522 (7)	
H11A	0.4144	−0.2567	0.4224	0.063*	
H11B	0.3154	−0.2875	0.3039	0.063*	
C12	0.2821 (3)	−0.2937 (2)	0.4645 (4)	0.0644 (9)	
H12A	0.2923	−0.2501	0.5346	0.077*	
H12B	0.2052	−0.2993	0.4178	0.077*	
C13	0.3272 (2)	−0.3936 (2)	0.5125 (3)	0.0426 (6)	
C14	0.4205 (3)	−0.4027 (2)	0.6155 (3)	0.0570 (8)	
H14	0.4556	−0.3463	0.6557	0.068*	
C15	0.4629 (3)	−0.4925 (3)	0.6604 (3)	0.0676 (9)	
H15	0.5260	−0.4965	0.7305	0.081*	
C16	0.4133 (3)	−0.5759 (3)	0.6031 (3)	0.0661 (9)	
H16	0.4424	−0.6369	0.6338	0.079*	
C17	0.3213 (3)	−0.5699 (2)	0.5008 (3)	0.0634 (9)	
H17	0.2874	−0.6270	0.4613	0.076*	
C18	0.2779 (2)	−0.4794 (2)	0.4550 (3)	0.0526 (7)	
H18	0.2149	−0.4760	0.3848	0.063*	
N4	0.30886 (18)	−0.14704 (16)	0.3532 (2)	0.0403 (5)	
O1	0.16542 (18)	−0.16633 (17)	0.16898 (19)	0.0610 (6)	
O2	0.43337 (15)	−0.08669 (16)	0.53428 (18)	0.0539 (5)	
Cl1	0.22370 (7)	0.21660 (6)	0.26068 (7)	0.0601 (2)	
Cl2	0.07822 (7)	0.20423 (6)	0.39214 (7)	0.0620 (2)	
Cl3	0.33930 (7)	0.15230 (6)	0.57446 (7)	0.0663 (3)	
Cl4	0.21264 (9)	−0.04238 (8)	0.62570 (8)	0.0806 (3)	
Cl5	0.01097 (7)	0.05813 (9)	0.13594 (8)	0.0834 (3)	
Cl6	0.00951 (8)	−0.10391 (8)	0.35359 (11)	0.0894 (4)	
H1	0.368 (2)	0.062 (2)	0.366 (2)	0.043 (7)*	
H2	0.232 (2)	0.0211 (19)	0.183 (2)	0.039 (7)*	

### Atomic displacement parameters (Å^2^)

**Table:**

	*U*^11^	*U*^22^	*U*^33^	*U*^12^	*U*^13^	*U*^23^
C1	0.0423 (13)	0.0338 (14)	0.0295 (11)	0.0036 (11)	0.0092 (10)	−0.0045 (10)
C2	0.0310 (12)	0.0318 (14)	0.0393 (13)	−0.0037 (11)	0.0152 (10)	−0.0033 (10)
C3	0.0329 (12)	0.0386 (15)	0.0460 (14)	0.0032 (11)	0.0188 (11)	0.0005 (12)
C5	0.0459 (14)	0.0467 (16)	0.0421 (14)	−0.0073 (13)	0.0280 (12)	−0.0097 (12)
C6	0.0377 (13)	0.0444 (15)	0.0294 (12)	−0.0014 (12)	0.0166 (10)	−0.0042 (11)
C7	0.0340 (12)	0.0560 (18)	0.0323 (12)	0.0029 (12)	0.0084 (10)	−0.0037 (12)
C8	0.0456 (15)	0.0471 (17)	0.0577 (17)	−0.0020 (13)	0.0336 (14)	−0.0047 (13)
C9	0.0601 (16)	0.0410 (15)	0.0392 (14)	0.0146 (13)	0.0323 (13)	0.0061 (12)
C10	0.0452 (14)	0.0411 (15)	0.0350 (13)	0.0101 (12)	0.0166 (11)	0.0044 (11)
C11	0.070 (2)	0.0319 (15)	0.0702 (19)	0.0018 (14)	0.0446 (17)	−0.0006 (14)
C12	0.072 (2)	0.051 (2)	0.091 (2)	0.0183 (17)	0.055 (2)	0.0242 (17)
C13	0.0465 (15)	0.0388 (15)	0.0510 (15)	0.0044 (13)	0.0279 (13)	0.0049 (12)
C14	0.0562 (18)	0.0514 (19)	0.0609 (19)	−0.0132 (16)	0.0182 (15)	−0.0101 (15)
C15	0.0499 (18)	0.081 (3)	0.063 (2)	0.0035 (19)	0.0097 (15)	0.0122 (19)
C16	0.075 (2)	0.051 (2)	0.083 (2)	0.0209 (19)	0.041 (2)	0.0135 (18)
C17	0.087 (2)	0.0437 (19)	0.071 (2)	−0.0118 (18)	0.043 (2)	−0.0190 (16)
C18	0.0504 (16)	0.066 (2)	0.0403 (14)	−0.0050 (15)	0.0150 (12)	−0.0024 (14)
N4	0.0498 (12)	0.0322 (12)	0.0466 (12)	−0.0012 (10)	0.0266 (10)	−0.0038 (10)
O1	0.0680 (13)	0.0623 (15)	0.0557 (12)	−0.0209 (12)	0.0260 (10)	−0.0277 (11)
O2	0.0415 (10)	0.0529 (13)	0.0573 (12)	0.0088 (10)	0.0061 (9)	0.0043 (10)
Cl1	0.0820 (6)	0.0462 (4)	0.0617 (5)	0.0098 (4)	0.0376 (4)	0.0159 (3)
Cl2	0.0724 (5)	0.0608 (5)	0.0613 (5)	0.0312 (4)	0.0343 (4)	0.0060 (4)
Cl3	0.0743 (5)	0.0543 (5)	0.0504 (4)	0.0050 (4)	−0.0010 (4)	−0.0221 (4)
Cl4	0.1149 (8)	0.0892 (7)	0.0602 (5)	0.0396 (6)	0.0587 (5)	0.0350 (5)
Cl5	0.0491 (4)	0.1271 (9)	0.0517 (5)	0.0178 (5)	−0.0085 (4)	−0.0190 (5)
Cl6	0.0718 (6)	0.0813 (7)	0.1426 (9)	−0.0319 (5)	0.0716 (6)	−0.0264 (6)

### Geometric parameters (Å, °)

**Table:**

C1—C9	1.514 (4)	C10—Cl2	1.762 (3)
C1—C10	1.547 (3)	C10—Cl1	1.769 (3)
C1—C2	1.554 (3)	C11—N4	1.464 (3)
C1—Cl3	1.751 (2)	C11—C12	1.515 (4)
C2—C3	1.512 (4)	C11—H11A	0.9700
C2—C6	1.535 (3)	C11—H11B	0.9700
C2—H1	0.91 (3)	C12—C13	1.507 (4)
C3—O2	1.202 (3)	C12—H12A	0.9700
C3—N4	1.381 (3)	C12—H12B	0.9700
C5—O1	1.200 (3)	C13—C14	1.375 (4)
C5—N4	1.387 (3)	C13—C18	1.384 (4)
C5—C6	1.514 (4)	C14—C15	1.366 (5)
C6—C7	1.552 (3)	C14—H14	0.9300
C6—H2	0.93 (3)	C15—C16	1.357 (5)
C7—C8	1.517 (4)	C15—H15	0.9300
C7—C10	1.548 (4)	C16—C17	1.357 (5)
C7—Cl5	1.742 (3)	C16—H16	0.9300
C8—C9	1.319 (4)	C17—C18	1.382 (5)
C8—Cl6	1.692 (3)	C17—H17	0.9300
C9—Cl4	1.688 (3)	C18—H18	0.9300
			
C9—C1—C10	99.5 (2)	C7—C10—Cl2	114.29 (18)
C9—C1—C2	107.2 (2)	C1—C10—Cl1	113.92 (18)
C10—C1—C2	101.12 (18)	C7—C10—Cl1	113.21 (17)
C9—C1—Cl3	116.46 (17)	Cl2—C10—Cl1	108.11 (15)
C10—C1—Cl3	115.44 (18)	N4—C11—C12	111.8 (2)
C2—C1—Cl3	115.00 (18)	N4—C11—H11A	109.3
C3—C2—C6	105.0 (2)	C12—C11—H11A	109.3
C3—C2—C1	113.8 (2)	N4—C11—H11B	109.3
C6—C2—C1	102.91 (19)	C12—C11—H11B	109.3
C3—C2—H1	110.1 (17)	H11A—C11—H11B	107.9
C6—C2—H1	113.8 (17)	C13—C12—C11	111.3 (2)
C1—C2—H1	111.1 (17)	C13—C12—H12A	109.4
O2—C3—N4	124.8 (3)	C11—C12—H12A	109.4
O2—C3—C2	127.3 (3)	C13—C12—H12B	109.4
N4—C3—C2	107.9 (2)	C11—C12—H12B	109.4
O1—C5—N4	124.7 (3)	H12A—C12—H12B	108.0
O1—C5—C6	127.8 (3)	C14—C13—C18	117.3 (3)
N4—C5—C6	107.5 (2)	C14—C13—C12	120.8 (3)
C5—C6—C2	105.2 (2)	C18—C13—C12	122.0 (3)
C5—C6—C7	114.7 (2)	C15—C14—C13	121.7 (3)
C2—C6—C7	103.08 (18)	C15—C14—H14	119.1
C5—C6—H2	107.6 (16)	C13—C14—H14	119.1
C2—C6—H2	114.6 (16)	C16—C15—C14	120.2 (3)
C7—C6—H2	111.6 (16)	C16—C15—H15	119.9
C8—C7—C6	106.8 (2)	C14—C15—H15	119.9
C8—C7—C10	99.4 (2)	C15—C16—C17	119.8 (3)
C6—C7—C10	101.1 (2)	C15—C16—H16	120.1
C8—C7—Cl5	117.48 (19)	C17—C16—H16	120.1
C6—C7—Cl5	115.15 (17)	C16—C17—C18	120.3 (3)
C10—C7—Cl5	114.6 (2)	C16—C17—H17	119.9
C9—C8—C7	107.7 (2)	C18—C17—H17	119.9
C9—C8—Cl6	128.2 (2)	C13—C18—C17	120.7 (3)
C7—C8—Cl6	123.8 (2)	C13—C18—H18	119.6
C8—C9—C1	107.5 (2)	C17—C18—H18	119.6
C8—C9—Cl4	128.4 (2)	C3—N4—C5	114.2 (2)
C1—C9—Cl4	123.8 (2)	C3—N4—C11	121.4 (2)
C1—C10—C7	92.4 (2)	C5—N4—C11	124.3 (2)
C1—C10—Cl2	114.45 (16)		

### Hydrogen-bond geometry (Å, °)

**Table:**

*D*—H···*A*	*D*—H	H···*A*	*D*···*A*	*D*—H···*A*
C2—H1···O2^i^	0.91 (3)	2.50 (3)	3.235 (3)	138 (2)

Symmetry codes: (i) −*x*+1, −*y*, −*z*+1.
